# Additive manufacturing of Al_2_O_3_ ceramics with MgO/SiC contents by laser powder bed fusion process

**DOI:** 10.3389/fchem.2023.1034473

**Published:** 2023-02-03

**Authors:** Asif Ur Rehman, Abid Ullah, Tingting Liu, Rashid Ur Rehman, Metin U. Salamci

**Affiliations:** ^1^ ERMAKSAN, Bursa, Türkiye; ^2^ Department of Mechanical Engineering, Faculty of Engineering, Gazi University, Ankara, Türkiye; ^3^ Additive Manufacturing Technologies Research and Application Center-EKTAM, Gazi University, Ankara, Türkiye; ^4^ CAS Key Laboratory of Mechanical Behavior and Design of Materials, Department of Modern Mechanics, University of Science and Technology of China, Hefei, Anhui, China; ^5^ School of Mechanical Engineering, Nanjing University of Science and Technology, Nanjing, Jiangsu, China; ^6^ Incheon National University, Incheon, South Korea; ^7^ Advanced Manufacturing Technologies Center of Excellence-URTEMM, Ankara, Türkiye

**Keywords:** additive manufacturing, sintering, defects, ceramic, oxidation, laser processing

## Abstract

Laser powder bed fusion is a laser-based additive manufacturing technique that uses a high-energy laser beam to interact directly with powder feedstock. LPBF of oxide ceramics is highly desirable for aerospace, biomedical and high-tech industries. However, the LPBF of ceramics remains a challenging area to address. In this work, a new slurry-based approach for LPBF of ceramic was studied, which has some significant advantages compared to indirect selective laser sintering of ceramic powders. LPBF of Al_2_O_3_ was fabricated at different MgO loads up to 80 wt%. Several specimens on different laser powers (70 W–120 W) were printed. The addition of magnesia influenced the microstructure of the alumina ceramic significantly. The findings show that when the laser power is high and the magnesia load is low, the surface quality of the printing parts improves. It is feasible to produce slurry ceramic parts without binders through LPBF. Furthermore, the effects of SiC and MgO loads on the microstructure and surface morphology of alumina are compared and analysed.

## Introduction

Complex shape ceramic components are manufactured utilizing a variety of traditional processes, such as hot isostatic pressing, extrusion, injection modelling, casting, and so on (Kingery, 1958). All conventional methods require more time and expensive tools such as drilling, milling gear shapers, and grinding machines to manufacture a part (Prakash et al., 2021). Additive Manufacturing (AM), widely known as 3D Printing, refers to a series of manufacturing methods in which parts and articles (3D objects) are made by layering materials (Redwood, 2018; Ullah et al., 2020; Ahmed et al., 2021). The fundamental working principles for additive manufacturing techniques are to add materials in a layer-by-layer fashion, however, the method with which materials are deposited and joined to make 3D objects differs between processes (Ansari et al., 2021; Kumar and Sharma, 2021; Mahmood et al., 2022). Laser powder bed fusion (LPBF), also widely recognized as selective laser melting (SLM), is a common laser-based AM technology that operates on the layer-by-layer manufacturing principle. LPBF can be used to fabricate three-dimensional ceramic parts directly, without using a sacrificial binder (Deckers et al., 2014a; Zhang et al., 2018; Aktrk et al., 2021; Rehman et al., 2021; Korkmaz et al., 2022a). This is the only direct method for printing pure ceramics. Other processes, including binder jetting, robocasting, and fused deposition of ceramics, are utilized to print ceramics, although these methods require binders or certain fibers to be added. Similarly, selective laser sintering (SLS) technology requires adding a binder to the raw material powder, while SLM can fabricate parts without utilizing any binders (Kruth et al., 2005). Direct printing of ceramics without using an external binder or additives is the key advantage of the SLM process. LPBF is preferred over other AM processes for a variety of reasons, including the feature that it is a “single-step” process that does not require any special post-processing processes. Throughout one-step powder bed fusion by complete melting, the laser beam causes the deposited material powder to heat and fully melt (Deckers et al., 2014b; Dezfoli et al., 2021a; Waqar et al., 2021a; Dezfoli et al., 2021b; Waqar et al., 2022). However, the whole system produces a very high-temperature gradient and residual stresses due to the rapid heating and cooling of printed layers (Sing et al., 2017; Waqar et al., 2021b; Korkmaz et al., 2022b; Khan et al., 2022). This is believed to be a severe issue for printing ceramics and composites due to their low thermal shock resistance (Zhang et al., 2022). Researchers have studied numerous individual aspects that affect the LPBF method, such as pre-heating, the surrounding temperature, scan speed, hatch distance, beam power, intervals duration, scanning strategy (orthogonal, islands, zigzag pattern, and many others), the effects of the non-steady-state melt regimes in the scanning tracks, the role of pores on crack initiation, laser pre-heating of ceramic material, powder particle density, and so on (Yamakov et al., 2002; Wilkes et al., 2013; Schwentenwein and Homa, 2015; Wang et al., 2017; Liu et al., 2020; Waqar et al., 2021b; Gokcekaya et al., 2021; Abdelmoula et al., 2022; Sun et al., 2022; Wang et al., 2022). Directly manufacturing highly dense ceramics with LPBF might result in fractures and other manufacturing flaws (Yamakov et al., 2002; WIPO, 2018). Abnormal grains form during the direct manufacturing of ceramics, resulting in severe component defects such as fractures and poor mechanical properties (Yamakov et al., 2002; Rahaman, 2017). In order to achieve a homogeneous and higher density, it is necessary to control abnormal grain growth. Therefore, further investigation of component defects and overcoming these defects is required. The addition of other materials to ceramic is one of the key approaches which can enhance manufacturability (Harun et al., 2012). The use of additives offers a very significant approach for fabricating ceramic with high density and controlled grain size structure (Rice, 2017). As MgO has a greater melting temperature (2,800°C–3,000 °C) than Al_2_O_3_, which has a melting temperature of 2,300 °C, it can increase manufacturability through crack deflection and pinning effect (Wu et al., 2001). Previous findings support that MgO enabled the ceramic structure become denser and enhancing physical and mechanical properties (Sathiyakumar and Gnanam, 2003; Harun et al., 2012).

Laser powder bed fusion of Alumina with Magnesia has tremendous opportunities and challenges as well. Composite materials consisting of a strengthening phase started developing very early. The reinforcements can be as particulate or as fibers. While these composite materials show great promise of improved strength and stiffness in the fabrication of parts. A preferable combination of several important properties such as high melting point, good thermal shock resistance, high resistance to chemical attack, high electrical resistivity, low thermal expansion coefficient, and potentially high mechanical strength at different temperatures, have made magnesium aluminate (MgAl_2_O_4_) very attractive for engineering applications (Stewart and Bradt, 1980; Baudin et al., 1995; Ganesh, 2013; Peng et al., 2021). Magnesium aluminate has drawn attention from the industries in the field of metallurgical, radio technical and electrochemical, chemical industries because of its excellent properties and environmental advantages (Peng et al., 2021). These functional properties make magnesium aluminate a good quality and superior variety of refractory material. It is also a commercially important ceramic reinforcement for metal matrix composites (MMC) fabrication because of its tailorable and functional properties. The Manufacturing of magnesium aluminate and the method for making refractories were available in the form of patents since 1905 (Sarkar, 2010). However, its higher cost of fabrication and production *via* traditional manufacturing techniques has limited its commercial acceptance.

LPBF offers the possibility of rapid manufacturing of ceramic composites which can have great promising applications unconstrained of their shapes. The studies were carried out using the eutectics phase diagram of alumina and magnesia as a reference (Callister, 2000). Magnesia is soluble in alumina at various temperatures and compositions. The solubility of a substance changes as its composition changes. Since solubility is affected by temperature, the same composition might have various levels of solubility at different temperatures. The purpose of this work is to examine how density and porosity changed as material proportions changed, in order to make dense pieces and reduced surface defects. The influence of LPBF process parameters and MgO content on the microstructure, density, and surface quality of Alumina parts are investigated in detail. The phase shift that occurs during the melting and sintering of the powder is also analyzed. In addition, the effects of SiC and MgO additions on the alumina microstructure and surface are analyzed and compared. This study will also investigate the optimal process parameters for Al_2_O_3_/MgO composite materials, in order to reduce major part defects and improve the overall ceramics manufacturing process using the LPBF technology.

## Experimental

### Materials and methods

**FIGURE 1:**
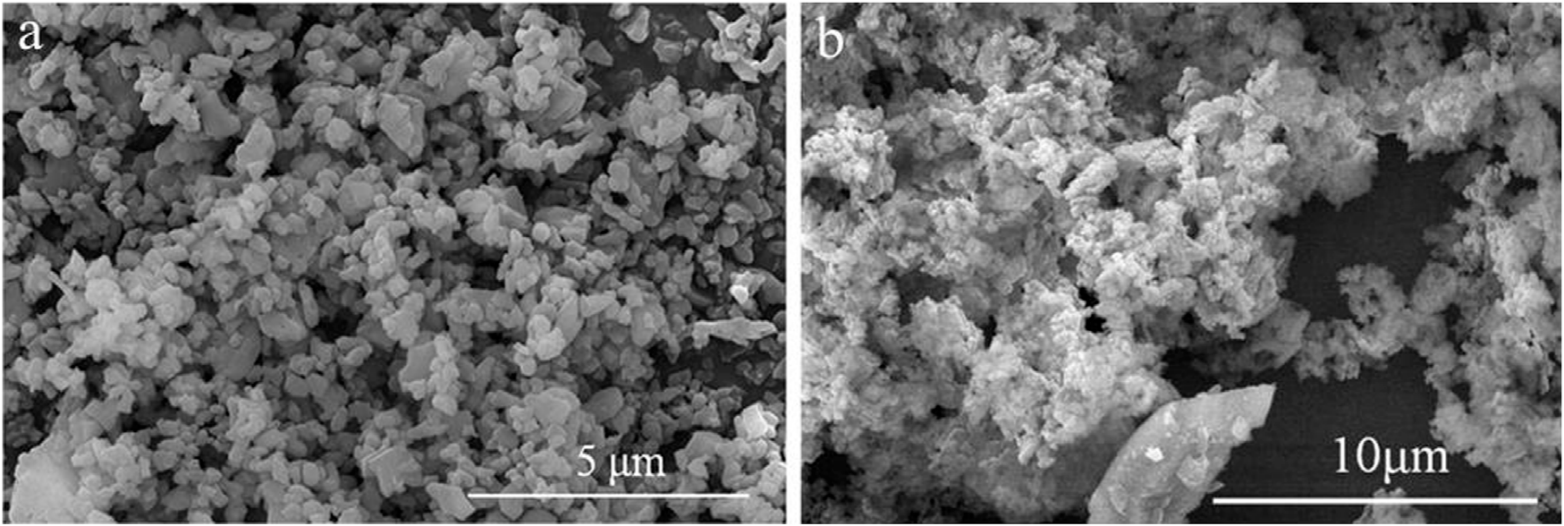
**(A)** Al_2_O_3_ powder **(B)** MgO powder (Zheng et al., 2019)

In this work MgO powders (Shanghai Chaowei Nanotechnology Co., Ltd.) of average particle size 1 μm and Al_2_O_3_ powder (produced by ALMATIS) of average particle size 0.4 μm with 99.9% purity were used for the experiments. The powders have irregular sheet structure, as shown in Figure 1. Several compositions with differing quantities of alumina and magnesia were sintered and melted. The experiments were performed with changing laser power while laser scanning speed and other process parameters such as hatch distance, layers thickness, and layers numbers were kept constant. Table 1 shows the experimental process parameters.

**TABLE 1:** Experimental process parameters.

Material (wt%)	Laser power W)	Scanning speed (mm/s)	Layer thickness (µm)	Hatch spacing (µm)	Scanning strategy
Al_2_O_3_: MgO = 20:80	70, 90, 110	200	50	50	Zigzag
Al_2_O_3_: MgO = 30:70					
Al_2_O_3_: MgO = 60:40					
Al_2_O_3_: MgO = 90:10					

### Layer deposition method

**FIGURE 2:**
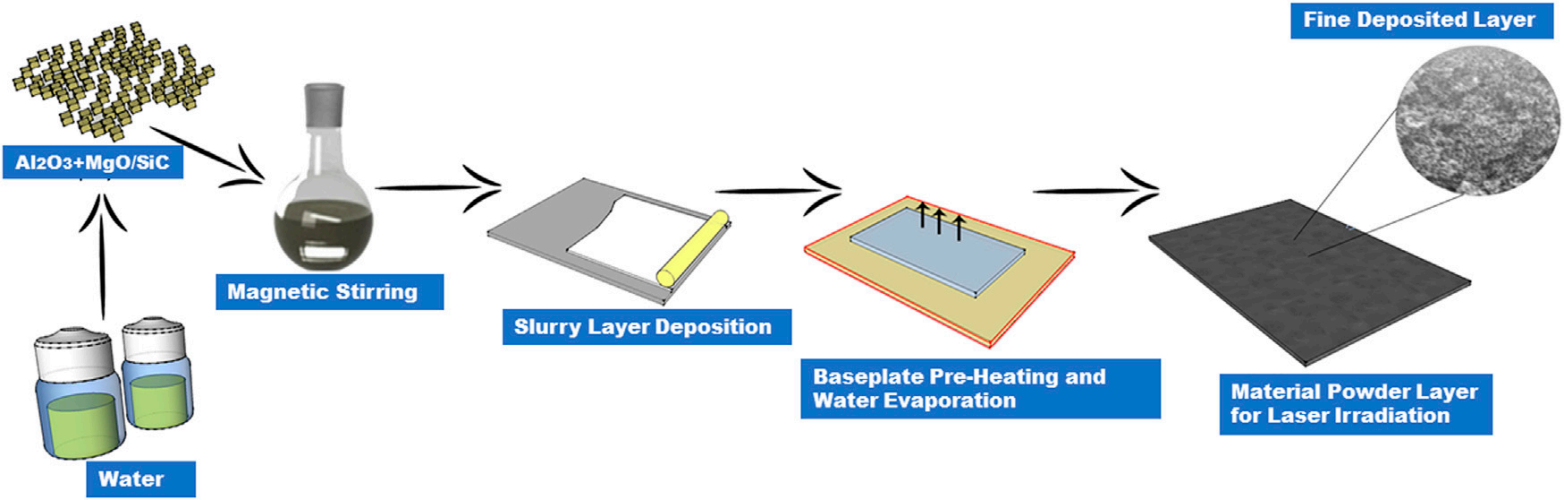
Layer deposition method.

**FIGURE 3:**
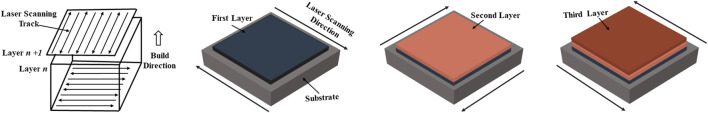
Laser scanning strategy and manufacturing method of the specimen.

Layer deposition of pure ceramics is difficult to maintain due to the inherent characteristics of ceramic powder. In this work, a two-step deposition method was used during the layer deposition process. Firstly, the powder was mixed with water as per the suitable material composition, resulting in a slurry that was deposited on the ceramic substrate fixed inside the layer moveable platform. The experimental platform has a powder leveling system, which is equipped with a rubber scraper. The scraper was used to spread and level the powder slurry in layers. The platform thickness of moveable systems can be extended from as smaller as 10 μm. The thickness of each layer was maintained as 50 μm. After the proper layer deposition, the water was fully evaporated by heating the base plate to 110 °C. The deposited layers appeared uniform when the water was evaporated, with no obvious deformities or differences between the top and bottom of the dried layers. The power layer was melted by directing the laser beam. The entire layer deposition process was repeated until the final part was produced with the required number of layers. Figure 2 illustrates the whole layer deposition process. The main objective of the slurry in this work was to make a homogeneous paste and appropriately deposit the layers according to the experimental requirements. The slurry approach was also used in the previous research for the manufacturing of alumina ceramic parts by digital light processing (DLP) based AM technique which plays an important role in the manufacture of ceramic parts (Zhang et al., 2022). Effective layer deposition requires appropriate slurry preparation, which has a significant impact on the surface morphology and the entire manufacturing process of ceramic products (Yamakov et al., 2002). Figure 3 shows the specimen’s 3D model and layers manufacturing process.

### Experimental setup

**FIGURE 4:**
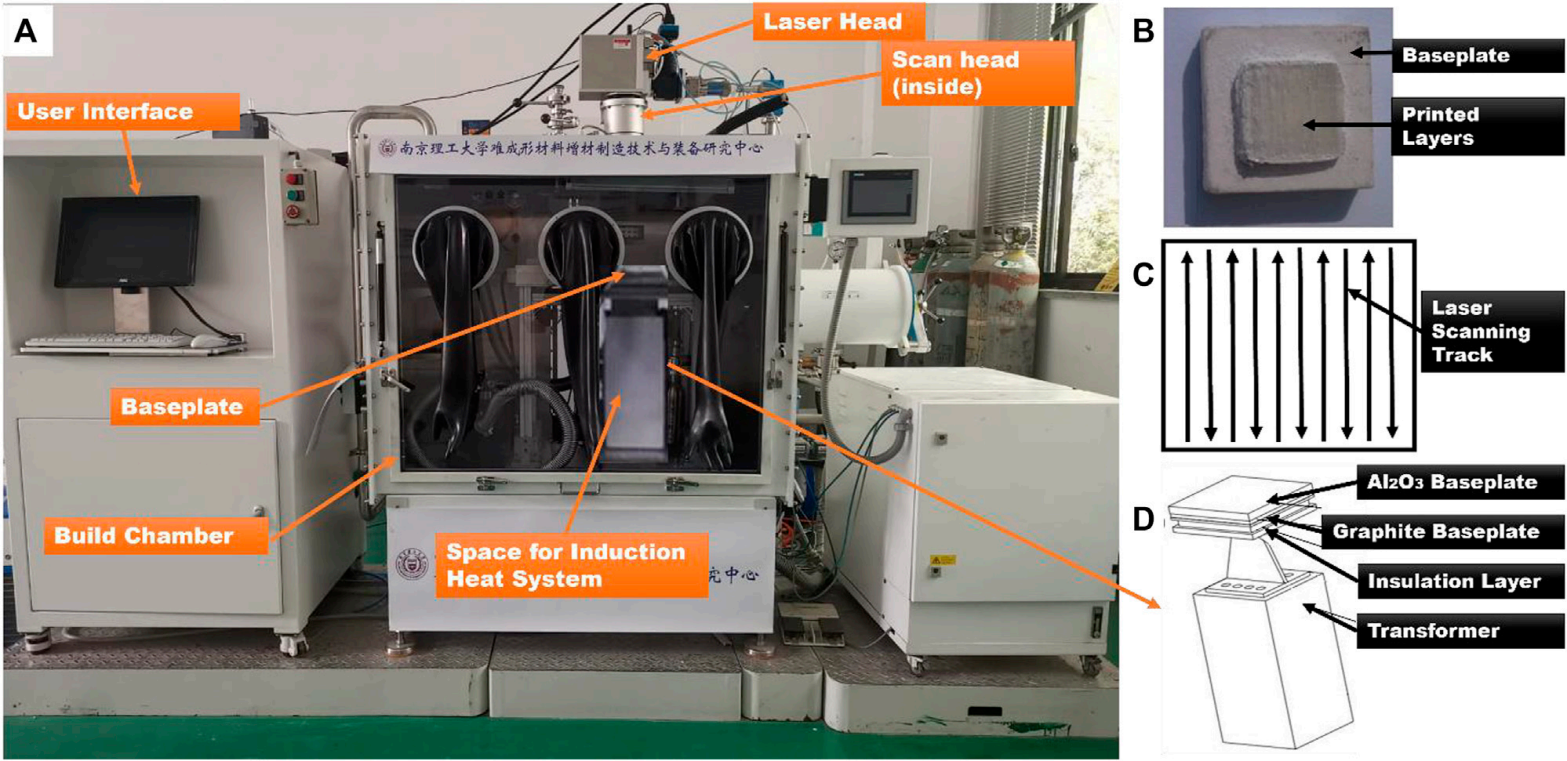
Experimental setup **(B)** Printed layers of ceramic specimen **(C)** Laser scanning strategy **(D)** Schematic of induction heating system.

The LPBF system is shown in Figure 4. The system is equipped with an IPG YLR-500 fiber laser, which creates a laser beam with a wavelength of 1.06 μm and can reach a maximum power of 500 W in continuous mode. The laser is led through a scanner (SCANLAB intelliSCAN 20). The spot size of the focused laser beam is about 60 μm. The system is also integrated with the induction heating system (20 KW) produced by the Shanghai Bamac capable of rapid heating, and the maximum preheating temperature is about 1,000 °C.

The tests were designed based on varying laser power (*p* = 50 W–100 W). The remaining parameters, such as laser scanning speed (200 mm/s), layer thickness (50 µm), laser hatch spacing (50 µm), and scanning strategy (zigzag), remained unchanged. Table 1 provides the experimental process parameters. The size of every part was kept under 25 layers.

### Measurements

Bruker D8 X-ray diffraction (XRD) was used to identify the structure, configuration, and quality of the samples. The XRD patterns were obtained at room temperature using Cu Kα radiation (*λ* = 1.5418Å) in continuous mode between 2θ = 10°–100°. The XRD was operated at tube current 40 mA and target voltage 40 kV at a scan speed of 2° min^-1^. Scanning Electron Microscopy (SEM) was used to analyze the surface morphology and microstructure of fabricated parts. SEM and EDS analysis (Oxford Instrument), the specimens were gold-coated (by the Leica ACE coater for 5 min).

## Results and discussions

### Materials aspect

**FIGURE 5:**
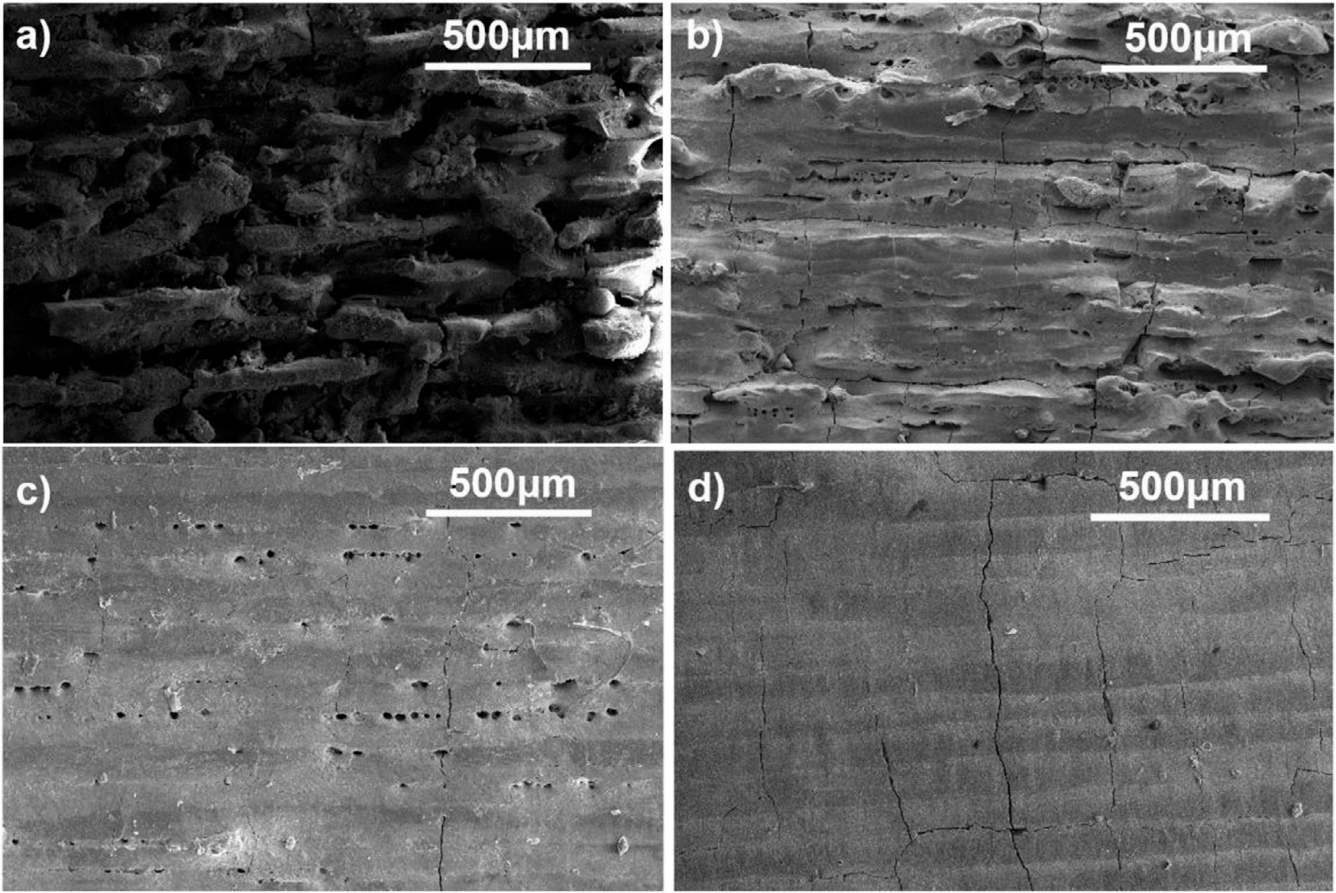
SEM **(A)** Alumina wt 20%: Magnesia wt 80% **(B)** Alumina wt 60%: Magnesia wt 40% **(C)** Alumina wt 75%: Magnesia wt 25% **(D)** Alumina wt 90%: Magnesia wt 10%, at laser power 70 W.

Scanning electron microscopy analysis of manufactured specimens using magnesia and alumina powders in various proportions revealed changes in density, porosity, and quality of the specimens produced. The effects of material change can be evaluated clearly as we increase the magnesia content, and the porosity increases at specific laser power (70 W). Increasing the magnesia content in material proportion with the decrease in alumina wt%. Similarly, increasing the alumina content and decreasing the Magnesia content causes a significant change in the surface quality and layers of the printed article, as shown in Figure 5. The sample fabricated from powders with high magnesium content (80%) shows poor surface quality, with obvious surface defects such as cracks and partially molten powder, as shown in Figure 5A. By reducing the magnesia content to 40% and increasing the alumina content to 60%, the surface quality of the part improved slightly. However, a large number of pores and cracks can still be seen, as shown in Figure 5B. The pores observed on the surface of the specimens are small spherical pores with identical sizes. Most of these pores are located on the edges of melting tracks, which can be controlled by an effective overlap of the neighbouring tracks. To restrict the formation of pores on the melting tracks’ edges, sufficient melting of the powder is required, as well as an efficient overlap of the adjacent tracks. The specimens made with a high alumina content show fewer surface defects and better melting conditions, which might be owing to alumina’s lower melting point and better melting behaviour compared to magnesia. The number of pores is highly controlled by lowering the percent weight of magnesia and increasing the quantity of alumina to 10% and 90%, respectively, as shown in Figure 5D. The experimental results show that if we desire a porous-free surface of alumina-magnesia composite, we should choose the maximum wt% of alumina. However, there seems to be no improvement in crack prevention or control, indicating that changing the material composition alone is insufficient to eliminate surface defects.

### Laser parameters aspect

**FIGURE 6:**
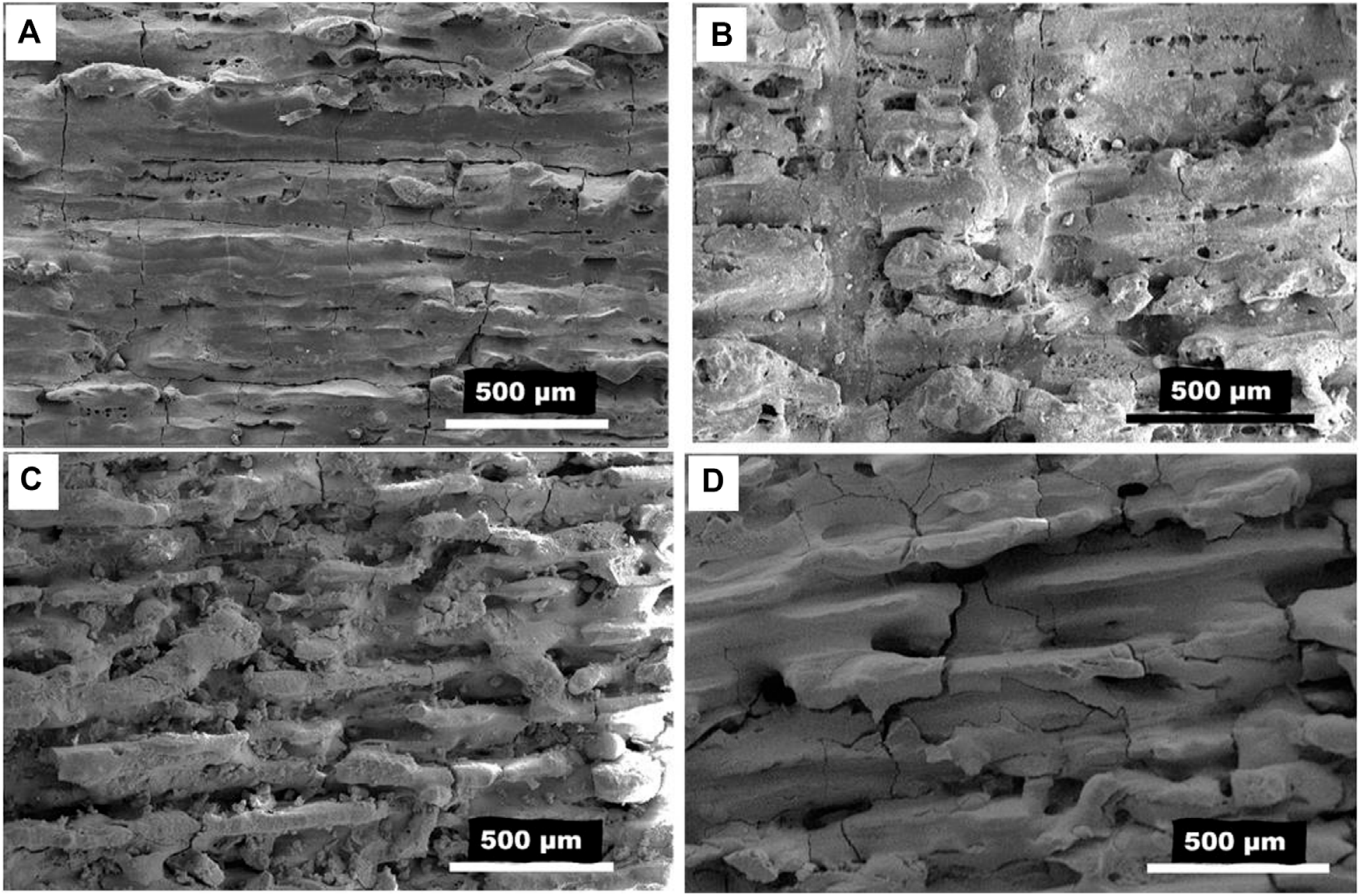
**(A)** Alumina wt% 60: Magnesia wt% 40 at Laser Power 70 W **(B)** Alumina wt% 60: Magnesia wt% 40, Laser Power 90 W **(C)** Alumina wt% 80: Magnesia wt% 20 at laser Power 70 W **(D)** Alumina wt% 80: Magnesia wt% 20 wt% at laser power 90 W.

The complete melting of powders is an important aspect of the LPBF process, which can eliminate several parts defects including cracks and porosity. To sufficiently melt the ceramic powders and improve the part’s manufacturability by reducing partly melted flaws, a higher laser energy input is required. A higher laser power within a specific range produced more energy input which can control numerous surface defects such as poor melting, fractures, and porosity. We keep the material composition constant and change the laser power, to study the change in the morphology of alumina and magnesia composites. The effects of laser power can be evaluated in Figure 6, as we decrease the laser power the porosity increases, and the density decreases similarly as we increase the laser power the porosity reduces and density increases. Figure 6A, B show the poor melting of the powder with pores observed on the surface of the samples. When the powder particles partly melt but do not fuse or merge into the melting pool, voids between the particles occur, which can lead to insufficient melting defects including cracks and poor density. The distribution pores and formation of microcracks are greatly reduced when the laser power is increased to 90 W, as seen in Figure 6B, D. Obtaining higher energy density by increasing laser power enhances not only the melting state of the material but also the fluidity of the laser melting tracks, which greatly enhances the bonding strength of the tracks. The stresses inside the layers are thus released more easily, which helps to reduce the driven force for crack growth (Zhang et al., 2022).

**FIGURE 7:**
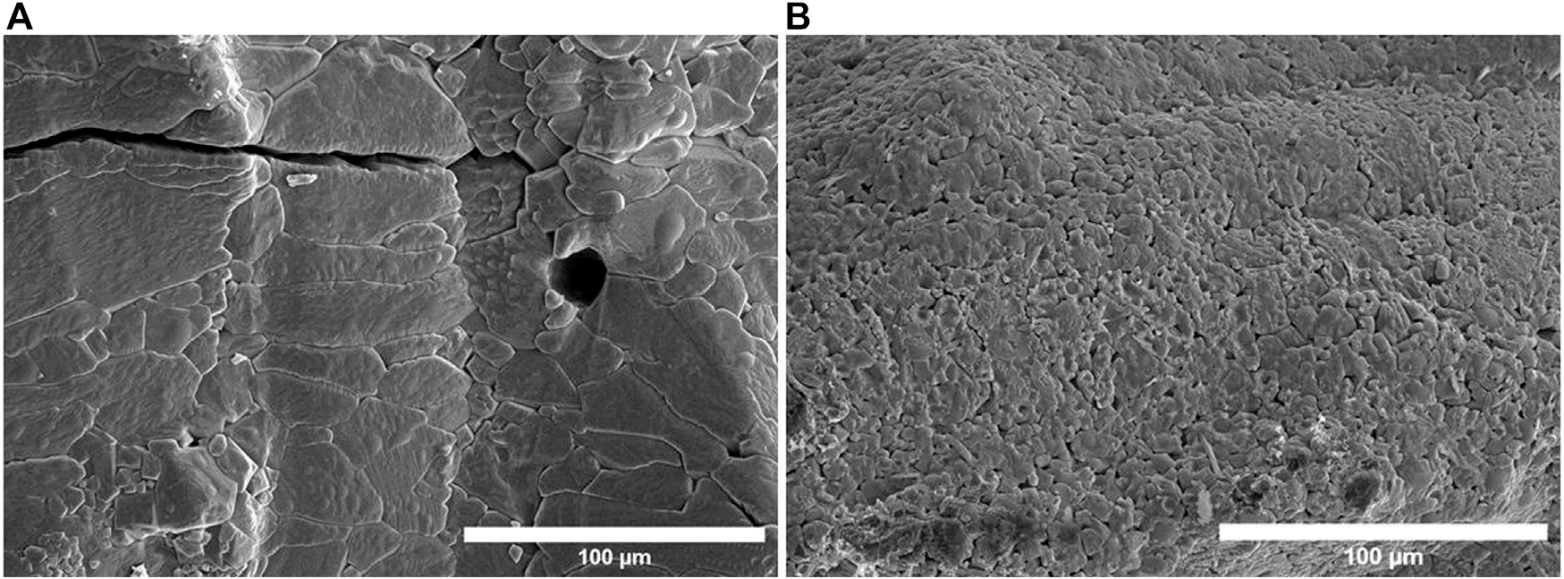
SEM Alumina-Magnesia Composite of 70 wt% Magnesia and 30 wt% alumina with Laser power **(A)** 50 W **(B)** 100 W.

**FIGURE 8:**
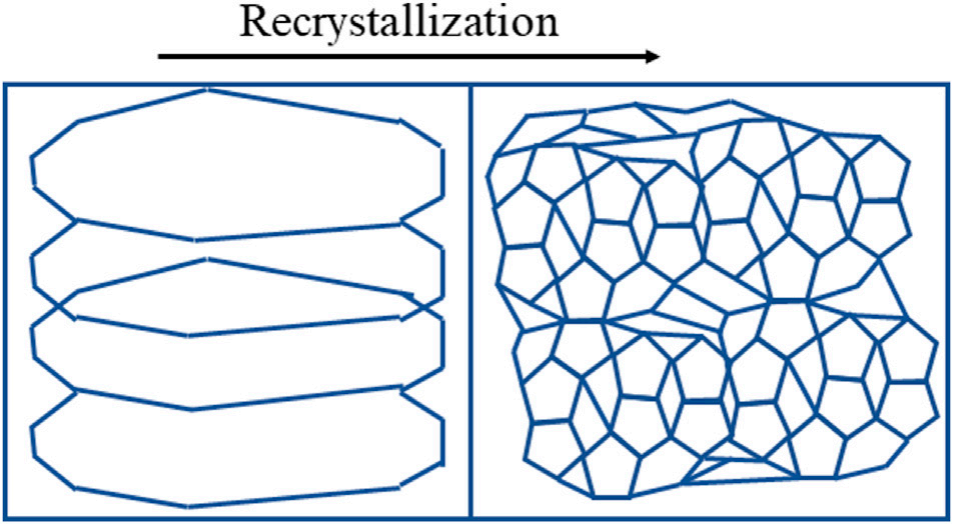
Schematic illustration of recrystallization by higher laser power.

Laser power has also a significant influence on the grain structure formation on the surface of alumina and magnesia composite. In most cases, problems arise when the laser heat input is too low or too high. Heat input that is too high, either through excessive laser power or slow scan speed, can slow the cooling rate, resulting in excessive grain growth. Previous studies have shown that the crystalline grains produced during the laser melting process are rapid to develop, complex, and tightly compacted, which results in part defects such as fracture junctions and shrinkage (Chen et al., 2019) (Ullah et al., 2020). Large and irregular grains may possibly appear with low energy input, especially when printing ceramic parts, as a result of insufficient powder melting, quick cooling, or an unstable microstructure (Ullah et al., 2022). Large grains can be observed in the sample produced with a lower laser power (50 W), which obviously subdivides and recrystallizes into small new grains by increasing the laser power to 100 W, as seen in Figure 7. The schematic illustration of grain recrystallization is shown in Figure 8. Grain growth is often undesirable in LPBF-manufactured components since it leads to several other defects. Previous research on the formation of grain structures shows that the grains produced by the laser melting process have several undesirable effects, including shrinkage and fractures (Chen et al., 2019). The size of cracks increases with the increase in grain size which may result in large fractures, as seen in Figure 7A). However, compared with coarse grains, small grains have some structural advantages as they increase the yield strength and macroscopic hardening of the material part (Yamakov et al., 2002).

### Comparison of SiC and MgO loads effect

**FIGURE 9:**
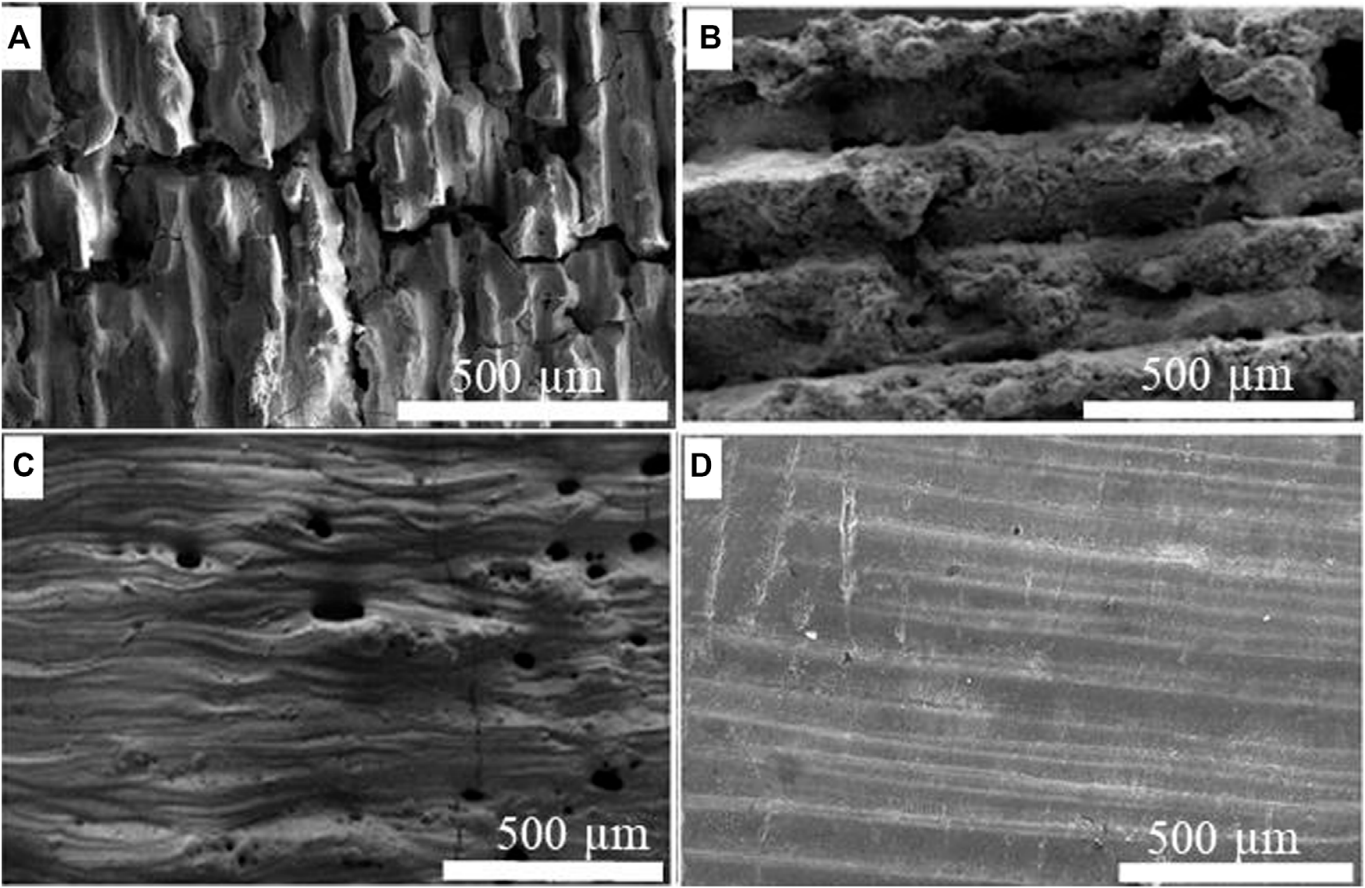
SEM of Al_2_O_3_/SiC samples at laser power 120 W **(A)** Al_2_O_3_ wt% 90, SiC wt% 10 **(B)** Al_2_O_3_ wt% 95, SiC wt% 5 **(C)** Al_2_O_3_ wt% 98, SiC wt% 2 **(D)** Al_2_O_3_ wt% 99.5, SiC wt% 0.5.

LPBF of alumina was also accomplished at various SiC loads up to 20 wt% which can be compared with the effect of MgO loads on alumina (Ur Rehman et al., 2022). The experimental results show that the influence of SiC on the microstructure and surface quality of alumina parts is relatively higher than the effect of MgO loads, as shown in Figure 9. When the SiC content was 10% or above, it initiated a chemical reaction between Al_2_O_3_ and SiC, causing structural and surface deformation, as seen in Figure 9A. This also resulted in excessive porosity and an undesirable appearance in the produced component. However, Figure 9C shows that when the amount of SiC was less than 5%, the microstructure improved significantly during PBSLP with no effect of LPS or chemical interaction. By further decreasing the content of SiC to 0.5 weight percent, a crack pinning impact could be clearly seen, as shown in Figure 9D.

The increase of MgO content seemed to have a similar effect on the microstructure and surface morphology of alumina. However, the experimental findings suggest that SiC content has a greater and more evident impact than MgO loading because the effect of MgO content is seen when the MgO content is only increased to 10% or above, as shown in Figure 5. This might be due to magnesia’s greater melting point and slower reaction rate than SiC with alumina. The impact of SiC loading, on the other hand, is only apparent when a very little quantity of SiC (less than 2%) is added to the alumina powder. The chemical reaction of SiC with Al_2_O_3_ is increased by adding further SiC (up to 20%), resulting in surface deformation and porosity. The higher interaction of SiC to metal oxides may also lead to high weight loss, and porosity and can disturb the melt pool and the surface morphology of the printing parts. The following reactions are found to occur when Al_2_O_3_ interacts with SiC.

$${SiC}_{\left(s\right)}+{Al}_{2}O_{3}\left(s\right)\to {Al}_{2}O_{\left(g\right)}+{SiO}_{\left(g\right)}+{CO}_{\left(g\right)}$$ (1)

$$2SiC\left(s\right)+{Al}_{2}O_{3}\left(s\right)\to {Al}_{2}O\left(s\right)\left(g\right)+2{Si}_{\left(l\right)}+2{CO}_{\left(g\right)}$$ (2)

$$3{SiC}_{\left(5\right)}+{Al}_{2}O_{3}\left(s\right)\to 2{Al}_{\left(l\right)}+3{Si}_{\left(l\right)}+3{CO}_{\left(g\right)}$$ (3)

### XRD analysis

**FIGURE 10:**
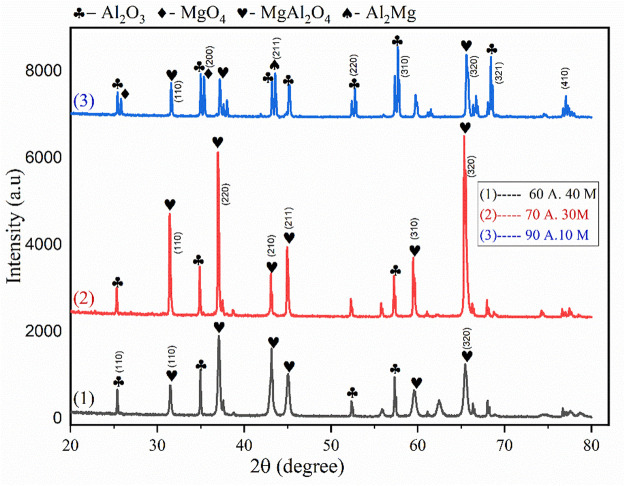
XRD of Al_2_O_3_-MgO specimens produced by LPBF process at laser power 100 W.

The XRD analysis shows that changing the material load has no obvious effect on the formation of MgAl_2_O_4_ composite, however, the density and overall microstructure of the composite are influenced. XRD peaks of the specimens formed with higher magnesia content (40wt%) show maximum peaks of MgAl_2_O_4_ with lower peak intensities of pure alumina, however, the sample produced of 30 wt% of magnesia shows higher peak intensities of MgAl_2_O_4_ with minimum peaks of MgO and pure alumina, as shown in Figure 10. In addition, the sample produced with higher Alumina content shows maximum peaks of Alumina with lower peak intensities of MgAl_2_O_4_ and MgO_4_. These findings show that samples produced of 30 wt% of Magnesia and 70 wt% of Alumina have maximum solubility of the powder and a more refined structure of MgAl_2_O_4_ composite which agrees with the Eutectic Phase diagram of Alumina-Magnesia (Callister and Rethwisch, 2011). The following possible chemical reactions may occur during the interaction of Al_2_O_3_ and MgO at high laser power. During the interaction of Al_2_O_3_ and MgO at high laser power, the following chemical reactions may occur.

$${Al}_{2}O_{3}\left(s\right)+MgO\left(s\right)\to {MgAl}_{2}O_{4}\left(s\right)$$ (4)

$$2{MgO}_{\left(s\right)}+3{Al}_{2}O_{3}\left(s\right)\to 2{MgO}_{4}\left(s\right)+3{Al}_{2}O_{\left(g\right)}$$ (5)

## Conclusion

The LPBF of Al_2_O_3_ was accomplished at different MgO loads up to 80 wt%. The microstructure of the composites was significantly affected by the addition of Magnesia. Samples with 30 wt% of magnesia show reduced part defects with maximum content and higher intensity peaks of MgAl_2_O_4_ composite. However, the higher magnesia loads (≥40%) show maximum parts defects and low solubility. Laser power has a significant influence on the surface morphology of the composite samples. The Surface quality of the samples becomes better when the laser power is higher and magnesia loads are lower. The effect of SiC and MgO loads on the fabrication of Al_2_O_3_ parts was compared, and it was found that silicon carbide loads are more effective than magnesia loads. Surface defects were decreased by the addition of lower (less than 2%) silicon carbide powder.

## Data Availability

The original contributions presented in the study are included in the article/supplementary material, further inquiries can be directed to the corresponding authors.
