# Corrigendum: Additive manufacturing of Al_2_O_3_ ceramics with MgO/SiC contents by laser powder bed fusion process

**DOI:** 10.3389/fchem.2023.1266823

**Published:** 2023-08-02

**Authors:** Asif Ur Rehman, Abid Ullah, Tingting Liu, Rashid Ur Rehman, Metin U. Salamci

**Affiliations:** ^1^ ERMAKSAN, Bursa, Türkiye; ^2^ Department of Mechanical Engineering, Faculty of Engineering, Gazi University, Ankara, Türkiye; ^3^ Additive Manufacturing Technologies Research and Application Center-EKTAM, Gazi University, Ankara, Türkiye; ^4^ CAS Key Laboratory of Mechanical Behavior and Design of Materials, Department of Modern Mechanics, University of Science and Technology of China, Hefei, Anhui, China; ^5^ School of Mechanical Engineering, Nanjing University of Science and Technology, Nanjing, Jiangsu, China; ^6^ Incheon National University, Incheon, Republic of Korea; ^7^ Advanced Manufacturing Technologies Center of Excellence- URTEMM, Ankara, Türkiye

**Keywords:** additive manufacturing, sintering, defects, ceramic, oxidation, laser processing

In the published article, there were errors in the **Funding** statement. The project name A2M2TECH was missing. An additional funding source, The Scientific and Technological Research Council of Türkiye, should also have been included. The correct Funding statement appears below:

“This work was supported by the Frontier Leading Technology Basic Research Project of Jiangsu (No. BK20202007), the National Natural Science Foundation of China (Grant No.52005262), BK20190463 and No. B18028. This work was also supported by the National Natural Science Foundation of China (grant number 51775281). This project has also received financial support from the European Union’s Horizon 2020 (H2020) research and innovation program under the Marie Skłodowska-Curie, grant agreement No. 764935. This work has also received funding from the H2020 research and innovation program under the Marie Skłodowska-Curie grant agreement No. 101034425 for the project titled A2M2TECH. This study has also received financial support from The Scientific and Technological Research Council of Türkiye (TUBITAK) with grant No 120C158 for the same A2M2TECH project under the TUBITAK’s 2236/B program.”

The authors apologize for these error and state that this does not change the scientific conclusions of the article in any way. The original article has been updated.

